# Pacing Lead-Induced Granuloma in the Atrium: A Foreign Body Reaction to Polyurethane

**DOI:** 10.1155/2013/396595

**Published:** 2013-05-29

**Authors:** Shinagawa Yoko, Yuka Kobayashi, Takao Iiri, Hitoshi Kitazawa, Masaaki Okabe, Hiroshi Kobayashi, Etsuo Okazaki, Yoshifusa Aizawa

**Affiliations:** ^1^Cardiovascular Center, Tachikawa General Hospital, Nagaoka 940-8621, Japan; ^2^Gastroenterology, Tachikawa General Hospital, Nagaoka 940-8621, Japan; ^3^Department of Pathology, Tachikawa General Hospital, Nagaoka 940-8621, Japan; ^4^Department of Research and Development, Tachikawa Medical Center, Tachikawa General Hospital, Nagaoka 940-8621, Japan

## Abstract

We described a case of an 82-year-old male who presented with a granuloma entrapping the polyurethane-coated pacing lead at the site of contact on the atrium. He had been paced for 8 years without symptoms or signs suggestive of an allergic reaction to the pacemaker system and died from thrombosis of the superior mesenteric artery and heart failure. A histological examination of the nodule showed an incidental granuloma with multinucleated giant cells. No granuloma was found in the heart or the lung.

## 1. Introduction

Allergic reactions to pacemaker compounds may occur rarely [1–3], and recognition of an allergic reaction is of vital importance to the pacemaker-dependent patient because total replacement of the pacemaker is the only effective therapy. In most cases, dermatitis is observed as the reaction to pacemaker, and the causal allergens were most commonly the metallic or plastic components [4–6].

The pacing lead is now coated by polyurethane that is considered to induce an allergic reaction in an extreme occasion [2]. Recently, we had a case of a patient with a pacemaker in which a granuloma was observed within a nodule which entrapped the polyurethane-coated pacing lead in the right atrium.

## 2. Case

The patient was an 82-year-old male. He underwent a colectomy for colon cancer at the age of 60. At the age of 74, a pacemaker was implanted for complete atrioventricular block (Generator: Nexus I Plus SR/3194, Ventricular lead: Thinline II/430-35S-58, tined-bipolar body 4.8 Fr, Intermedics Inc., St. Paul, MN, USA) and had been paced on VVIR mode. Diabetes mellitus was pointed out at that time. At the age of 80, he underwent a surgery for dissecting aneurysm of the ascending aorta and was complicated by cerebral infarction. However, he had been uneventful thereafter. On 16 June 2011, he developed nausea, tarry stool, and dyspnea and was admitted to our hospital.

On admission, he weighed 75 kg and was 165 cm in height. His body temperature was 36.5°C. His pulse rate and blood pressure were 83 beats per min and 109/73 mmHg, respectively. A physical examination was noncontributory. Oxygen saturation was 85%, and CRP was elevated to 5.0 mg/dL. HbA1c was 5.5%. Otherwise, the laboratory examination was normal. No eosinophila was found in the complete blood counts.

### 2.1. Course during Hospitalization

An emergency endoscopic examination revealed multiple ulcers in the descending colon, and he was diagnosed to have ischemic enteritis. Biopsy showed no malignancy. Following heparin and warfarin administration, the lesion improved to normal. Meanwhile, he developed increasing dyspnea and pulmonary congestion. He was treated by furosemide and human atrium natriuretic peptide, and the cardiothoracic ratio decreased from 58% to 50%, and his symptom disappeared.

On the 12th day of hospitalization, he developed severe abdominal pain in the right side of abdomen. The abdominal CT suggested superior mesenteric artery (SMA) obstruction. However, surgery was not accepted by the patient and his family because of high age, and he received only supportive therapy. The patient died two days later.

### 2.2. Autopsy

Autopsy revealed total occlusion of the SMA with fresh thrombi and massive intestinal necrosis, but the original ischemic lesion of the descending colon was improved to normal. The lungs and liver were congested, and the coronary arteries showed diffuse and severe stenosis at multiple sites with mural thrombi and multiple areas of infarct.

**Figure 1:**
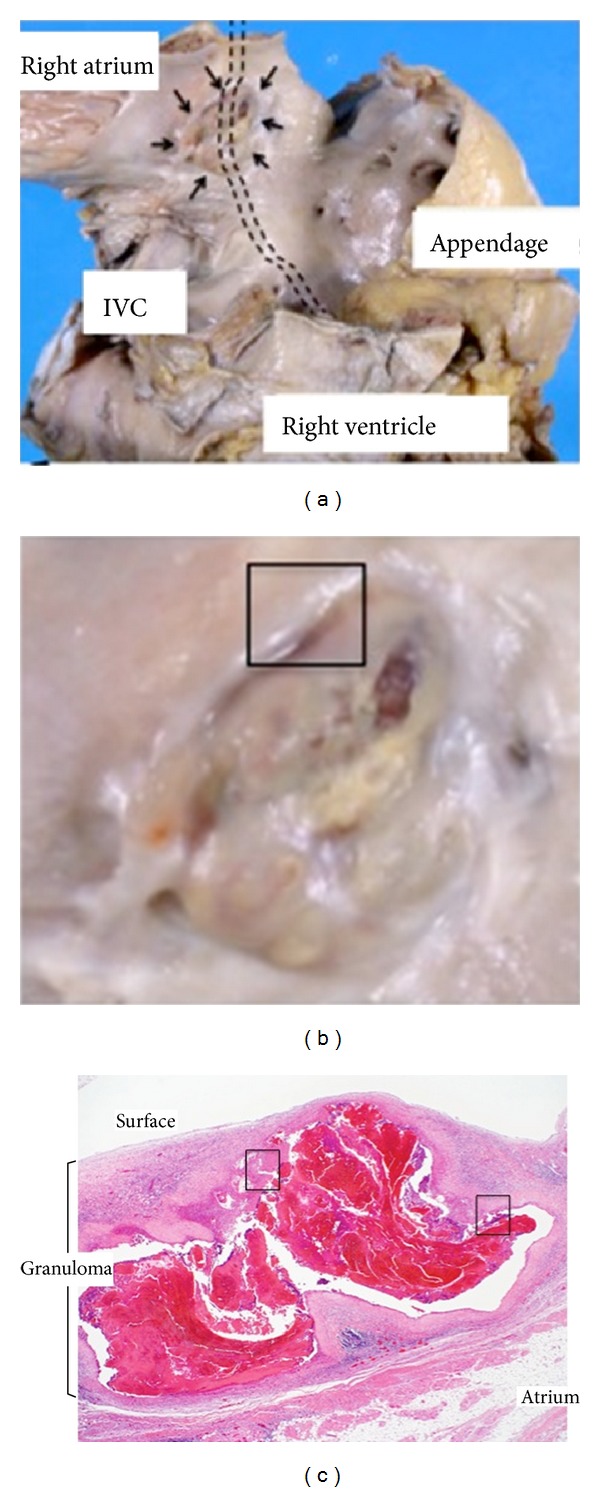
Macro-and microscopic findings. (a) At autopsy, the pacemaker lead was entrapped by a nodule. After detachment of the lead from the nodule, the base of the nodule was 1.5 × 1.0 cm in size as shown by the arrows. The possible course of the lead was depicted by dotted lines. (b) The nodule had a broad basis on the endocardium of the right atrium. The edge was cut and used for microscopic examination (rectangle). (c) Histologically, the nodule revealed fibrosis, lymphocyte infiltration, small vessels, and hemorrhagic lesions.

**Figure 2:**
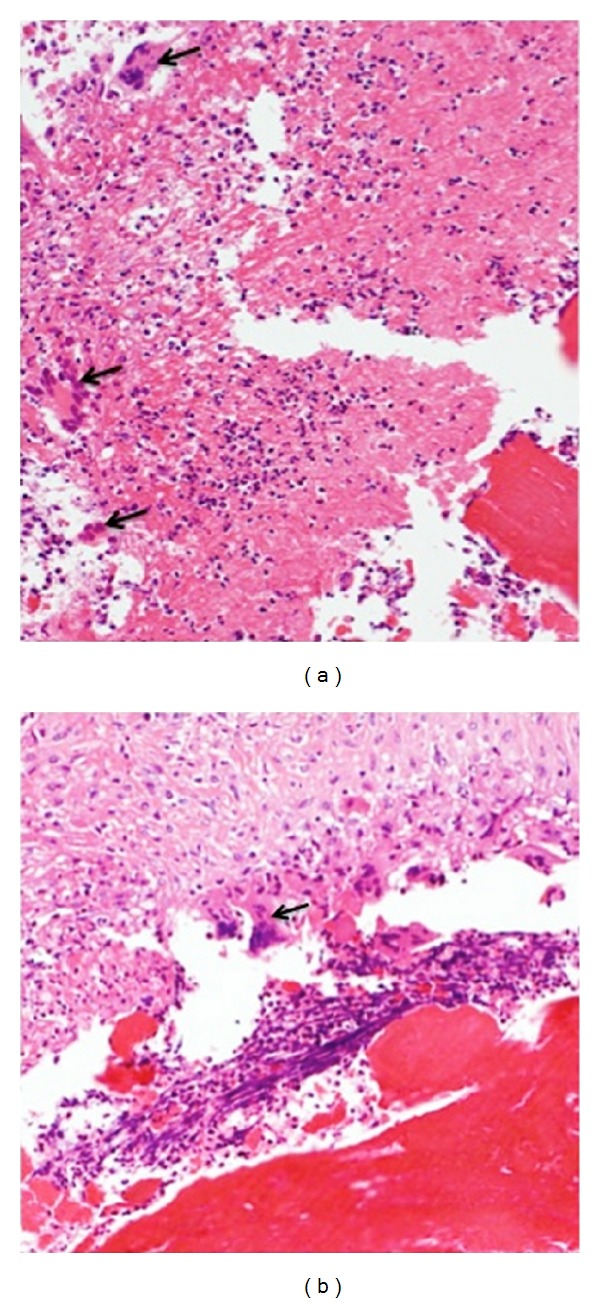
Multinuclear giant cells. In the two regions denoted by rectangles in Figure 1(c), multinuclear giant cells are observed as shown by arrows ((a) and (b)).

The pacing lead coated by polyurethane (80 A) was entrapped by a nodule of 1.5 × 1.1 cm in size in the right atrium. The nodule was located at the contact site of the lead on the endocardium at the lateral site of the right atrium (Figure 1(a)). Histologically, the nodule consisted of fibrosis and thrombi. It contained amorphous eosinophilic material and multinucleated foreign body-type giant cells (Figure 2). There was no granulomatous lesion in other organs including the heart, the lung, or at the sites of adhesion within the vein. No microorganisms were found.

## 3. Discussion

The patient had been under VVIR pacemaker therapy for 8 years and died from SMA occlusion and heart failure. He revealed no evidence of an allergic reaction, locally or systemically. At autopsy, he was found to have a nodule around the pacing lead within the atrium. Histologically, multinucleated giant cells were observed in the nodule. There was no granulomatous lesion in other organs, and the granuloma was considered to be unrelated to the present illness.

Allergic reactions to pacemaker system are one of serious complications [3], and to avoid allergic reaction, the pacemaker system is now coated by polyurethane [1–4]. Polyurethane was reported to induce foreign body reactions only rarely, but granuloma in the capsules surrounding the polyurethane-coated implants [7–10].

In the cardiac devise, a pacemaker-related granuloma has been reported to occur adjacent to the lead-electrode parts of a permanent pacemaker [11–13]. The patient had been under pacing for a long time suggesting the possibility that the granuloma is a reaction to the foreign body of pacemaker.

A granuloma was reported in a patient under pacemaker therapy around the infected epicardial lead [14], and to our knowledge, this is the first case of intracardiac granuloma formed around the polyurethane-coated pacing lead. The patient revealed no evidence of an allergic reaction, locally or systemically, and a granuloma with multinucleated giant cells was observed incidentally at autopsy in the atrium. The granuloma can be a result of mechanical irritation of the pacing lead on the endocardium of the atrium for a long time.

Its clinical implication was not apparent, but a possible relation to occurrence of pulmonary embolism is to be studied.
